# Troubles du sommeil, symptômes anxio-dépressifs et risque cardio-vasculaire chez les hypertendus noirs africains: étude transversale de 414 hypertendus suivis en ambulatoire au CHU de Ouagadougou (Burkina Faso)

**DOI:** 10.11604/pamj.2015.21.115.5219

**Published:** 2015-06-12

**Authors:** Nobila Valentin Yaméogo, André Samadoulougou, Larissa Justine Kagambèga, Aimé Arsène Yaméogo, Eric Ilboudo, Georges Millogo, Jonas Kologo, Jean Yves Toguyeni, Jacques Simporé, Patrice Zabsonré

**Affiliations:** 1 Service de Cardiologie, CHU Yalgado Ouédraogo, Ouagadougou, Burkina Faso; 2 Service de Cardiologie, CHU Sanou Souro, Bobo Dioulasso, Burkina Faso; 3 Centre de Recherche Biomoléculaire, Ouagadougou, Burkina Faso

**Keywords:** Troubles du sommeil, anxiété, dépression, risque cardio-vasculaire, HTA, noir, africain, Sleep disorders, anxiety, depression, cardiovascular risk, HBP, black, african

## Abstract

**Introduction:**

Dans le but de déterminer la fréquence des troubles du sommeil, de l'anxiété et de la dépression, et de rechercher l'existence d'une relation entre ces troubles et le risque cardio-vasculaire global chez les hypertendus noirs africains, nous avons réalisé une étude transversale de mai 2010 à mars 2011 à l'unité de consultation externe du service de cardiologie du CHU-Yalgado Ouédraogo de Ouagadougou qui a inclut 414 hypertendus adultes suivis en ambulatoire. Après un examen clinique à la recherche des facteurs de risque cardio-vasculaire, deux auto-questionnaires ont été administrés.

**Méthodes:**

Le questionnaire de l’«European Sleep Center » pour la recherche des troubles du sommeil et, l’échelle «Hospital Anxiety and Depression Scale » de Zigmond et Snaith pour la recherche de l'anxiété et la dépression. Le diagnostic du syndrome d'apnée du sommeil était clinique et basé sur la présence des 4 symptômes principaux: hypersomnolence diurne, éveils nocturnes fréquents avec nycturie, asthénie matinale avec ou sans céphalées et ronflements importants. Le risque cardio-vasculaire global était calculé grâce à l’équation d'Anderson tirée de l’étude de Framingham. L'analyse des données a été réalisée par le logiciel SPSS version 17. La comparaison des variables a été effectuée grâce au test de Khi 2 pour les variables qualitatives et au test «t» de Student pour les variables quantitatives. Le seuil de signification a été fixé à 5%.

**Résultats:**

L’échantillon était composé de 414 patients dont 248 femmes (59,9%). L’âge moyen était de 54,6 ± 9,3 ans. Les troubles du sommeil étaient retrouvés dans 72,2% des cas. Ils étaient dominés par l'insomnie (49,2%), le syndrome d'apnée du sommeil (33,5%) et le syndrome des jambes sans repos (25,8%). L'anxiété était retrouvée dans 37,1% des cas et la dépression dans 16,6% des cas. Le risque cardio-vasculaire global était faible dans 21,0%, modéré dans 33,6%, élevé dans 24,4% et très élevé dans 21,0% des cas. Le syndrome d'apnée du sommeil (p=0,033), le syndrome des jambes sans repos (p=0,005), l'anxiété (p=0,0021) et la dépression (p=0,0001) étaient significativement associés à un risque cardio-vasculaire élevé à très élevé.

**Conclusion:**

Les troubles du sommeil, l'anxiété et la dépression sont fréquents chez les hypertendus noirs africains. Ils sont liés à un niveau de risque cardio-vasculaire élevé. Leur dépistage devrait être intégré à la prise en charge des hypertendus.

## Introduction

L'hypertension artérielle constitue un véritable problème de santé publique. Son étiopathogénie reste obscure mais plusieurs facteurs organiques, psychologiques, environnementaux et la qualité du cycle veille-sommeil sont incriminés dans l'apparition et le pronostic de cette affection [1]. Parmi ces facteurs, les troubles du sommeil, l'anxiété et la dépression occupent une place importante [2–4]. Les objectifs de ce travail était de déterminer la fréquence des troubles du sommeil, de l'anxiété et de la dépression, et de rechercher l'existence d'une relation entre ces troubles et le risque cardio-vasculaire global d'une population d'hypertendus noirs africains suivis en ambulatoire.

## Méthodes

Du 1^er^ Mai 2010 au 31 Mars 2011, nous avons inclus de manière consécutive 414 hypertendus adultes suivis en ambulatoire à l'unité de consultation externe du service de cardiologie du CHU-Yalgado Ouédraogo de Ouagadougou (Burkina Faso). Une fiche épidémiologique a permis de recueillir les paramètres sociodémographiques (âge, sexe, lieu de résidence, niveau scolaire, statut marital, profession, niveau socio-économique), les antécédents (familiaux psychiatriques, personnels psychiatriques et médico-chirurgicaux), les habitudes de vie (tabac, alcool, sédentarité), les paramètres cliniques (tension artérielle systolique (TAS), tension artérielle diastolique (TAD), indice de masse corporelle), paracliniques (la glycémie à jeûn, la créatininémie, l'uricémie, le lipidogramme et l’électrocardiogramme) et les paramètres thérapeutiques (durée du traitement antihypertenseur, nombre d'antihypertenseurs). Deux auto-questionnaires ont été ensuite simultanément administrés: l'un pour l’évaluation de l'anxiété et de la dépression selon l’échelle «Hospital Anxiety and Depression Scale » de Zigmond et Snaith (HAD) [5]. Il s'agit d'un auto-questionnaire mixte, composé de 14 items: 7 items évaluant l'anxiété (définie par un score supérieur à 11) et 7 autres évaluant la dépression (définie par un score supérieur à 11). La cotation se fait par paliers de 0 à 3. Le score global HAD supérieur à 19 indique la présence d'une dépression majeure; l'autre, le questionnaire de l’ «European Sleep Center », pour la recherche des troubles du sommeil. Les troubles du sommeil recherchés étaient les dyssomnies (insomnie, trouble du cycle circadien, narcolepsie, syndrome des jambes sans repos) et le syndrome d´apnée du sommeil. Le diagnostic du syndrome d'apnée du sommeil était basé sur la présence des 4 symptômes principaux: hypersomnolence diurne, éveils nocturnes fréquents avec nycturie, asthénie matinale avec ou sans céphalées et ronflements importants [6]. Le risque cardio-vasculaire était calculé en référence à l’équation d'Anderson tirée de l’étude de Framingham [7]. Les patients souffrant d'hypertension artérielle secondaire n’étaient pas éligibles. L'analyse des données a été réalisée par le logiciel SPSS version 17. La comparaison des variables a été effectuée grâce au test de Khi 2 pour les variables qualitatives et au test de Fisher pour les variables quantitatives. Le seuil de signification a été fixé à 5%.

## Résultats

Les questionnaires ont été administrés à 414 patients.

**Caractéristiques générales de la population**: le sex-ratio était de 1,4 en faveur des femmes. L’âge moyen était de 54,6 ± 9,3 ans (extrêmes de 32 et 76 ans). Plus de la moitié de nos patients (53,3%) habitaient Ouagadougou et sa banlieue. Les caractéristiques générales de la population sont résumées dans le Tableau 1.

**Tableau 1:** Caractéristiques générales de la population des 414 hypertendus

Caractéristiques	Nombre	Pourcentage
Age (moyenne et écart-type)= 54,6 ± 9,3 ans		
Sexe masculin	166	40,1
Sexe féminin	248	59,9
Mariés	399	96,3
Résidence Ouaga et sa banlieue	367	88,6
Durée du traitement ≥ 3 ans	303	73,1
Niveau socio-économique faible	288	69,5
Non scolarisés	244	58,9
Hypertrophie ventriculaire gauche électrocardiographique	208	50,2
Chiffres tensionnels élevés (PAS ≥ 140et/ou PAD ≥ 90mmHg)	201	48,5
Traitement associant au moins 3 antihypertenseurs	176	42,5
Cultivateurs et femmes au foyer	137	33,1
Commerçants	106	25,6
Secteur informel	103	24,8
Fonctionnaires	68	16,4

**Les troubles du sommeil**: les troubles du sommeil étaient retrouvés dans 72,2% des cas. Ils étaient dominés par l'insomnie (49,2%), le syndrome d'apnée du sommeil (33,5%) et le syndrome des jambes sans repos (25,8%). La distribution des troubles du sommeil dans la population figure sur le Tableau 2.

**Tableau 2:** Distribution des troubles du sommeil dans la population de 414 hypertendus

Trouble	Nombre	Pourcentage
Insomnie	204	49,2
Syndrome d'apnée du sommeil	139	33,5
Syndrome des jambes sans repos	107	25,8
Narcolepsie	66	15,9
Trouble du cycle circadien	60	14,4

**Les symptômes anxio-dépressifs**: la sommation des items de l’échelle «Hospital Anxiety and Depression Scale » de Zigmond et Snaith a permis de conclure à une anxiété chez 154 patients soit 37,1% des cas et une dépression chez 69 patients soit 16,6% des cas. L'anxiété était significativement plus fréquente chez les femmes (p=0,002), les non scolarisés (0,006), les patients ayant un niveau socio-économique bas (0,012) et ceux présentant une insomnie (0,004). Quand à la dépression, elle était plus fréquente chez les hommes (p=0,047), les fonctionnaires (p=0,002), les patients présentant un syndrome des jambes sans repos (p= 0,0011) et ceux ayant un syndrome d'apnée du sommeil (p=0,0041).

**Le risque cardio-vasculaire**: les facteurs de risque cardio-vasculaire outre l'hypertension artérielle étaient dominés par l’âge (≥45 ans pour l'homme et ≥55 ans pour la femme) (63,0%), le sexe masculin (40,1%), les dyslipidémies (29,7%) et le diabète (23,9%). Les principaux facteurs de risque cardio-vasculaire sont résumés dans le Tableau 3. Le risque cardio-vasculaire global calculé selon la méthode de Framingham était faible dans 21,0%, modéré dans 33,6%, élevé dans 24,4% et très élevé dans 21,0% des cas. La relation entre les troubles du sommeil, les symptômes anxio-dépressifs et le risque cardiovasculaire globale est présentée dans le Tableau 4. Le syndrome d'apnée du sommeil, le syndrome des jambes sans repos, l'anxiété et la dépression étaient significativement plus retrouvés chez les patients dont le risque cardio-vasculaire est élevé à très élevé.

**Tableau 3:** Distribution des facteurs de risque cardio-vasculaire chez les 414 hypertendus

Facteur de risque	Nombre	Pourcentage
Age (≥ 45 ans pour l'homme et ≥55 ans pour la femme)	261	63,0
Sexe masculin	166	40,1
Dyslipidémie	123	29,7
Diabète	99	23,9
Surcharge pondérale/Obésité	94	22,7
Sédentarité	52	12,5
Tabagisme	29	7,0

**Tableau 4:** Relation entre trouble du sommeil, symptômes anxio-dépressif et risque cardiovasculaire

Troubles	Risque faible à modéré n=226	Risque élevé à très élevé n= 188	p
Insomnie	48,2%	50,5%	0,054
Syndrome d'apnée du sommeil	30,0%	32,9%	**0,033**
Syndrome des jambes sans repos	21,2%	31,3%	**0,005**
Narcolepsie	18,1%	13,2%	0,772
Trouble du cycle circadien	16,8%	11,7%	0,671
Anxiété	29,2%	46,8%	**0,0021**
Dépression	09,3%	25,5%	**0,0001**

## Discussion

Le diagnostic du syndrome d'apnée du sommeil était purement clinique, sans polysomnographe. Cette méthode pourrait surestimer la proportion des patients souffrant de ce trouble du sommeil. Elle constitue une limite de notre étude.

### Troubles du sommeil, hypertension artérielle et morbimortalité cardio-vasculaire

Notre étude montre une fréquence élevée des troubles du sommeil chez les hypertendus (72,2%). Ces troubles du sommeil augmentent la morbimortalité cardio-vasculaire [8–12]. L'hypertension artérielle semble associée à une courte durée de sommeil (cinq heures ou moins) [13]. Le déficit de sommeil (insomnie) induit un processus inflammatoire et un stress oxydatif qui peuvent expliquer les relations entre insomnie et maladies cardio-vasculaires [14]. L'insomnie était le trouble le plus fréquent dans notre étude (49,2%). Le syndrome d'apnée du sommeil est une cause majeure d'hypertension artérielle [15]. Il s'agit aujourd'hui d'un problème reconnu de santé publique de par sa fréquence et la somnolence associée [16], la morbidité cognitive [17] et cardio-vasculaire [18, 19], particulièrement l'hypertension [20, 21], l'insuffisance coronaire [22], les accidents vasculaires cérébraux [23], les troubles du rythme [24] et enfin la mortalité [25] qui lui sont attribués. Dans notre étude, sa fréquence était de 33,5%. Le syndrome des jambes sans repos est un trouble fréquent dont la prévalence est estimée à environ 10% de la population adulte [26]. Ce syndrome est responsable d'insomnie et de somnolence diurne. Sa présence est liée à un développement de l'hypertension artérielle. Ce trouble a été objectivé dans 25,8% des cas dans notre série. La narcolepsie était retrouvée dans l'ordre de 16% dans notre étude. Très peu de publications concernent ce sujet probablement en raison du faible nombre de patients souffrant de ce trouble. Des relations entre poids, obésité et narcolepsie sont suggérées sans que le mécanisme en cause ne soit clairement identifié [27]. Il est probable que ce soit un ensemble de facteurs (somnolence, réduction de l'activité physique, traitements médicamenteux, déficit en orexine, etc.) qui entraîne la prise de poids observée chez les patients narcoleptiques [27]. Cette prise de poids augmente naturellement le risque cardio-vasculaire.

### Symptômes anxio-dépressifs

L'anxiété était fréquente dans notre série (37,1%). Ce résultat est proche de celui trouvé par J. Masmoudi et al. dans une population d'hypertendus tunisiens [28]. Pour certains auteurs, la dépression est un facteur de risque voire un facteur étiologique dans la survenue des facteurs de risque cardio-vasculaire, tels que le diabète, l'HTA, etc. Des études ont en effet montré un lien étroit entre des scores élevés de dépression et l'augmentation du risque cardio-vasculaire [29, 30]. Dans notre étude les symptômes dépressifs ont été retrouvés dans 16,6% des cas.

### Le risque cardio-vasculaire global

Nous avons trouvé une relation étroite entre syndrome d'apnée du sommeil (0,033), syndrome des jambes sans repos (0,005), anxiété (0,0021) et dépression (0,0001), et le risque cardio-vasculaire élevé. Ces données corroborent celles de la littérature. Elles contribuent à l'augmentation du niveau du risque cardio-vasculaire en intervenant par plusieurs mécanismes: la genèse de facteurs de risque, l'induction d'un processus inflammatoire et d'un stress oxydatif, l'inobservance thérapeutique et l'aggravation des facteurs de risque déjà existants.

## Conclusion

Cette étude montre que les troubles du sommeil et les symptômes anxio-dépressifs sont fréquents chez les hypertendus noirs africains. Le cardiologue doit par conséquent s'intéresser à ces troubles et les rechercher de manière systématique. Leur présence présage d'un risque cardio-vasculaire élevé. Il convient outre mesure de redorer le tri-axe neurologie-psychiatrie-cardiologie pour leur meilleure prise en charge.
